# The Mental Well-Being of Health Care Workers during the Peak of the COVID-19 Pandemic—A Nationwide Study in Poland

**DOI:** 10.3390/ijerph18116101

**Published:** 2021-06-05

**Authors:** Mateusz Babicki, Ilona Szewczykowska, Agnieszka Mastalerz-Migas

**Affiliations:** 1 Department of Family Medicine, Wroclaw Medical University, 51-141 Wroclaw, Poland; agnieszka.mastalerz-migas@umed.wroc.pl; 2 Intensive Care Unit, Wroclaw Medical University, Borowska Street 213, 50-556 Wroclaw, Poland; ilo.sz@wp.pl

**Keywords:** mental health, second wave of pandemic, COVID-19, GHQ-28, anxiety

## Abstract

Introduction: The current epidemiological situation has quickly led to several changes in the daily functioning of people around the world, especially among medical personnel, who in this difficult period were burdened with new professional duties, which significantly affects their mental health. Materials: This study aims to assess the mental health of health professionals at a critical point in their workload, to compare the results with those the general population, and to explore the potential determinants affecting it. The CAWI survey includes a sociodemographic section, work experience and a standardised psychometric tool (GHQ-28). Data were collected during the second wave of the COVID-19 pandemic in Poland (3–29 November 2020), which had the highest mortality rates and SARS-CoV-2 morbidity rates, as well as during the period of a significant increase in deaths, compared to the corresponding pre-pandemic period. Results: A total of 2150 surveys were eligible for analysis. Among them, 848 (39.4%) were active health professionals. In the analysis of the scores of the GHQ-28 scale and its sub-scales, evaluating anxiety/insomnia and somatic symptoms, medical workers scored significantly higher scores than non-medical professions (*p* < 0.001). Frontline medical workers (*p* < 0.001) and those who were forcibly seconded to work with COVID-19-infected patients (*p* = 0.011) achieved significantly higher GHQ-28 scores. Conclusions: The COVID-19 pandemic has had a significant impact on mental deterioration among health professionals, especially among those directly working with SARS-CoV-2-infected patients and those who were forcibly seconded to work with such patients. To mitigate the effects of the pandemic, appropriate psychological care for medical personnel needs to be implemented.

## 1. Introduction

In a short time, the current epidemiological situation has led to several changes in the daily functioning of people all over the world [1]. Due to the rapid spread of SARS-CoV-2, in less than four months after the first report concerning the unidentified pathogen, WHO declared a pandemic state, resulting in numerous restrictions [2,3]. COVID-19, caused by the SARS-CoV-2 infection, is a disease with a very broad spectrum of symptoms. In the vast majority of cases, the symptoms are completely asymptomatic or mildly symptomatic. According to current reports, however, nearly 5% of patients may have a severe course of the disease and 14% a moderate course. Such patients often require hospitalisation [4]. The rapidly increasing number of new infections led to a situation where at one moment the number of individuals requiring medical intervention, including hospitalisation, was very high. That situation significantly affected the functioning of health facilities that were already heavily burdened [5]. Furthermore, the healthcare system is extremely underfunded and it is facing staff shortages at every level of healthcare. According to OECD (Organisation for Economic Co-operation and Development), government expenditure on healthcare in Poland is only 6.5% of GDP and is one of the lowest in the European Union; the EU average is 9.8% of GDP [6].

The increasing numbers of new cases, rising mortality rates, lack of therapeutic options, heavy workload, lack of adequate access to personal protective equipment, and lack of support are determinants that might adversely affect the mental health of medical personnel [7]. Experience shows that pandemic states create a growing need to increase the number of health professionals or force them to be more professionally engaged and to work beyond their limits under tremendous pressure due to both the fear for their own health and the fear of possibility of infecting their loved ones [8]. Which can translate into an enormous psychological burden, especially among medical staff, whose mental and physical well-being is key to the further fight against the pandemic.

Past experience concerning the 2003 SARS outbreak shows that health professionals, especially those directly involved in fighting the disease, experienced the psychological effects of their work [9,10]. Early evidence of the COVID-19 fight confirms those reports, especially among medical personnel directly involved in the COVID-19 fight. Currently, as in the past, attention should be given to the early health assessment of the medical personnel. In addition, the implementation of comprehensive measures to improve their health status should be considered [7,11,12,13].

This study aims to assess and compare the COVID-19 preventive behaviours and the prevalence rate of mental disorders in medical and non-medical professionals in Poland based on a nationwide survey. The secondary objective of this study is to assess how both direct COVID-19 patient care and forced secondment of medical personnel to fight the pandemic affect COVID-19-preventive behaviours, as well as the prevalence rate of mental disorders. As part of the study, the following hypotheses were proposed: (1) the COVID-19 pandemic significantly affected the mental health of both medical and non-medical professionals; (2) medical workers are more favourably disposed to preventive behaviours and compliance with government recommendations than other professions; (3) medical professionals working with COVID-19 patients exhibit poorer mental health; (4) forced secondment to fight the COVID-19 pandemic significantly worsens mental health; (5) gender, psychiatric past, chronic conditions and economic instability have an impact on medical staff mental health.

## 2. Materials and Methods

### 2.1. Methods

The Computer-Assisted Web Interview (CAWI) survey, as a voluntary, fully anonymous questionnaire distributed online via a social network, was addressed to medical and non-medical professionals, aged 18 or older, who at the time of the survey were staying in Poland. The questionnaire was distributed via social media, and also among groups whose membership is only for healthcare professionals, which is checked by group administrators, and joining them requires a license number to practice. The respondents were informed about the study objectives, methodology, and estimated duration before they were allowed to take part in the survey. After the respondents were familiarised with the information, they gave their informed consent to participate in the survey. When completing the questionnaire, the respondents were given the opportunity to opt out of the survey without giving any reason.

The data collection period (3–29 November 2020) in Poland correlated with the highest COVID-19-related morbidity rates, daily mortality rates (number of COVID-19 cases: min. 19.15, max. 27.87) and deaths caused by COVID-19 (min. 92, max. 637). This led to rapid changes in daily social life and a sudden increase in workload for medical personnel [14]. Moreover, according to Statistics Poland, the total number of deaths during the study period was nearly 60,000, while in the corresponding 2019 period the number was twice lower [15].

The presented study was approved by the Bioethics Committee of the Wroclaw Medical University (approval number: KB-471/2020) and it was conducted in accordance with the Declaration of Helsinki.

The proprietary questionnaire consisted of questions assessing the sociodemographic status of the respondents, including their age, sex, place of residence, marital status, educational level, past medical history of somatic and psychiatric conditions. The above-mentioned questionnaire also contained questions that verified profession. In the case of health professionals, those questions addressed their COVID-19-related work experience and their voluntariness regarding working with COVID-19 patients. To assess attitudes towards the COVID-19 pandemic, the respondents were asked if they limited their gatherings with family, friends, as well as their outings, due to the ongoing situation. In each case, the following answers were possible: I strongly agree/I agree/I neither agree nor disagree/I disagree/I strongly disagree. The level of anxiety of the respondents was assessed by the questions “On a scale of 1–10 (1 being no anxiety, 10 being extreme anxiety), how serious is your fear of COVID-19 infection?” The second question was similar but it referred to the concern for a loved one.

The next part included the GHQ-28 questionnaire, which is a common tool used for assessing mental disorders. The GHQ-28 questionnaire is based on a four-point Likert scale (0 being not at all, 1 being no more than usual, 2 being rather more than usual, 3 being much more than usual). The maximum possible number of available points to score was 84. The cut-off score of clinical significance was 24 points [15]. Its interpretation can also be carried out at the level of subscales that included individual questions covering relevant somatic symptoms (items 1, 3, 4, 8, 12, 14 and 16), anxiety and insomnia (items 2, 7, 9, 13, 15, 17 and 18), social dysfunctions (items 5, 10, 11, 25, 26, 27 and 28) and severe depression (items 6, 19, 20, 21, 22, 23 and 24) [16,17]. The overall internal consistency of the scale in this study, based on Cronbach’s alpha, was 0.939.

The original questionnaire is presented in the supplementary material (Survey).

### 2.2. Participants

As many as 2155 respondents participated in the survey. Five individuals did not give their consent to participate. A total of 2150 correctly completed surveys were eligible for further analysis.

The exact profile of the surveyed group is shown in Table 1. The survey involved 848 (39.4%) active health professionals. Doctors, numbering 649 (76.5%), and nursing staff, 97 (11.4%), were the most numerous groups among health professionals. The other medical staff were: dentist (2.3%), paramedic (1.1%), laboratory diagnostician (0.6%), X-ray technician (0.2%) and others (7.9%). A total of 561 (66.2%) surveyed medical professionals worked directly with COVID-19 patients, of which 38 (4.8%) worked in a dedicated COVID-19 hospital, 108 (12.7%) in a COVID-19 unit in a non-dedicated COVID-19 hospital, 341 (40.2%) in a primary care clinic, and 74 (8.7%) in both a hospital and a primary care clinic. Seventy-four (9.7%) medical workers were seconded to work with COVID-19 patients, of which 63 were seconded by the decision of the health facility management and 11 by the decision of national authorities.

### 2.3. Statistical Analysis

The analysis was performed using STATISTICA 13.0, StatSoft (StatSoft, Hamburg, Germany). The analysed data are both quantitative and qualitative. Descriptive statistics were used for descriptive purposes. Qualitative variables were compared using the chi-squared test. The normality of distribution for the variables was evaluated by means of three different statistical tests: the Kolmogorov–Smirnov test, Lilliefors test, and Shapiro–Wilk W test with the significance level of *p* = 0.05. Due to lack of fulfilment the criterion of normality of distribution differences, the Mann–Whitney U test and the Kruskal–Wallis test were used. Initially, the authors analysed the GHQ-28 scale, each of its subscales, the level of fear of being infected with COVID-19 and the level of fear for the health of loved ones. COVID-19 preventive behaviours, predispositions to obey the guidelines concerning wearing protective masks, and a subjective assessment of mental health between health professionals and other professionals were also analysed. An analogous comparison was shown based on the division of health professionals into those who worked directly with COVID-19 patients and those who were forcibly seconded to fight the COVID-19 pandemic. Doctors were compared with other medical professions. An analysis of covariance (ANCOVA) was then performed to assess the differences between medical and non-medical professionals while taking into account the potential confounding factors (age, sex, place of residence, educational level, limited earning capacity). The results were analysed by means of post-hoc tests using the Tukey’s test. The statistical significance level of *p* < 0.05 was established at each stage of the survey.

## 3. Results

### 3.1. Comparison of Mental Health and COVID-19 Preventive Behaviours among Medical and Non-Medical Professionals

Detailed comparative data between medical and non-medical professionals are shown in Table 2.

In the comparative analysis between professions, statistically significant differences were not observed; only in terms of social dysfunction and severe depression. Medical workers predominate in both the total GHQ-28 score and the percentage of positive scale scores.

The level of fear of being infected with COVID-19 is significantly higher among medical personnel; 25.94% of them fear contracting COVID-19 more than other diseases, compared to 16.67% of non-medical professionals. The medical professionals achieved a mean score higher by 0.69 (*p* < 0.001) on the linear scale for the level of fear of contracting COVID-19. In the case of the concern for the health of loved ones, their mean score was higher by 1.18, (*p* < 0.001). Both medical and non-medical professionals show significantly higher levels of concern for the health of their loved ones than for their own health.

In each of the above-mentioned situations concerning the limitation of interpersonal contacts, a significantly higher reduction of them is observed in medical workers. Similarly, they have more positive attitudes towards the obligation to cover the nose and mouth in both indoor (*p* < 0.001) and open (*p* < 0.001) spaces.

In a subjective assessment of the effect of the pandemic on mental health, as many as 72.28% of medical workers described it as negative and only 0.9% stated it was positive.

### 3.2. A Detailed Analysis of Individual Subgroups among Health Professionals

Table 3 shows in detail the effect of direct work with COVID-19 patients and forced secondment to fight the pandemic. The forced secondment of medical professionals to fight the COVID-19 pandemic significantly exacerbates their psychotic symptoms, expressed in higher values on the GHQ-28 scale, excluding the subscale assessing social dysfunction.

When doctors were compared with other medical groups, they had statistically higher scores on each of the GHQ-28 subscales.

In the case of health professionals, 76.42% search the web daily for information concerning COVID-19 and 66.51% track daily statistics concerning the number of COVID-19-related deaths and cases. Information seeking is related to higher GHQ-28 scale scores (30.46 vs. 27.31, *p* < 0.001) as well as subscales assessing anxiety and insomnia (9.67 vs. 8.11, *p* < 0.001), and somatic symptoms (7.66 vs. 6.32, *p* < 0.001). Similarly, tracking statistics is correlated with higher GHQ-28 scale scores (30.21 vs. 27.77, *p* < 0.001) as well as subscales assessing anxiety and insomnia (9.53 vs. 8.35, *p* < 0.001), and somatic symptoms (7.52 vs. 6.58, *p* < 0.001).

### 3.3. Analysis of Demographic Factors on the Mental Condition of Health Care Workers

The impact of sociodemographic variables on the mental condition is presented in Table 4.

In overall interpretation, as well as in each of the GHQ-28 subscales, women obtained significantly higher scores than men (*p* < 0.001). The restriction on earning opportunities during the COVID-19 pandemic was significantly associated with the feeling of anxiety/insomnia severity among the medical staff.

### 3.4. Analysis of Potencial Confounding Factors

The analysis of covariance, ANCOVA, did not prove that the distribution of sex, age, marital status, earning capacity during the pandemic, past psychiatric history, chronic medical conditions, quarantine or COVID-19 infection in the respondent and their family member statistically significantly affected the differences between medical and non-medical professionals on the GHQ-28 scale. The subscale assessing somatic symptoms proved a correlation between the limitation of earning capacity: F 2.548; *p* = 0.002 and the anxiety subscale in relation to sex: F 5.155; *p* = 0.023.

## 4. Discussion

Since the end of 2019, when the first viral pneumonia of SARS-CoV-2 aetiology was diagnosed, daily functioning of, in particular, health professionals has been rapidly modified. This has a significant impact on their mental health [18]. The above-mentioned impact was confirmed in numerous reports from, for example, China, Nepal, Italy and France [19,20,21,22,23].

The results of this study correlate with the previous reports, and they clearly indicate that medical workers are significantly more mentally strained during the COVID-19 pandemic than workers from other professions. Among other things, a study conducted among Italian medics indicates that people in direct contact with patients suffering from COVID-19 show higher depressive symptoms (*p* = 0.005, *d* = 0.40) and PTSS (*p* = 0.015, *d* = 0.47) than medics not working with COVID-19 [19,20,21,22,23]. In their subjective assessment of own mental health, 72.27% of medical workers indicated that their mental health was impaired, compared to 63.90% of other professionals. In the analysis of the psychometric tool, GHQ-28, the said correlation was confirmed and as many as 64.27% of medical workers scored at least 24 points (mean 30.35 ± 14.53), indicating the presence of mental disorders. Furthermore, medical workers had significantly higher scores on subscales concerning anxiety, sleep disorders and somatic symptoms. A study conducted in Nepal on 475 health care workers indicates that anxiety disorders during the pandemic period occurred in 41.9%, depression in 37.5% and sleep disorders in 33.9% of them, which indicates a significant psychological burden and is consistent with the results of this report [23]. This significant increase in mental disorders may be due to public pressure to fight COVID-19 and the heavy workload of medical personnel. According to the World Bank in Poland, the number of hospital beds per 1000 inhabitants in Poland is 6.5. This is a much lower number than in other European countries [24]. The ratio of nurses and doctors per 1000 inhabitants is 2.4 and 5.1, respectively. This is well below the EU average [25,26]. In the face of a massive influx of patients requiring medical intervention and staff shrinkage due to COVID-19 morbidity, the workload of health professionals was enormous and frequently beyond their limits.

Although a high ratio was observed in both analysed groups on the linear scale of fear for one’s own health and that of loved ones, health professionals rated their fear significantly higher, especially in terms of the fear for the health of their loved ones, obtaining an average score of 8.67 vs. 5.79 on a ten-point scale. The results are consistent with studies conducted in eight European countries (Austria, Germany, Switzerland, Italy, France, Spain, Portugal, England), where medical personnel showed higher fear for the health of their loved ones than for their own health, averaging 2.25 points SD 0.82 in the case of concern for health families, compared with 1.12 points SD 1.04, based on a four-point Likert scale [27]. This was also confirmed in a previous pandemic among nursing staff in Thailand [28]. Both qualitative and cross-sectional studies carried out so far showed that the fear of COVID-19 infection—one’s own or of loved ones—is one of the strongest determinants of mental health deterioration. Moreover, prolonged emotional tension can significantly contribute to the accumulation of psychopathological symptoms [29]. It should be also noted that work under chronic stress can significantly lead to disruption of the functioning of the hypothalamic-pituitary-adrenal (HPA) axis, and thus increase the risk of developing psychopathological changes [30,31,32]. Work during the COVID-19 pandemic is a chronic stressor, especially during the period with the highest morbidity and mortality rates so far, and with a nearly double total number of deaths compared to the pre-year period [14,15].

Nevertheless, medical workers seem to be more adapted to the new reality. For that reason, they showed a much higher acceptance of minimising both interpersonal contacts with family/friends and outings as one of the options to fight the pandemic. This may be caused by greater awareness of the threat posed by SARS-CoV-2. As previously proved, the level of knowledge concerning the COVID-19 disease plays an important role in the response to an epidemic crisis [33]. These attitudes, by limiting gatherings with family and friends, may lead to an increasing longing for face-to-face meetings with loved ones, which significantly affects mental health. On the other hand, however, high levels of anxiety and fear of infection increase medical workers’ sense of responsibility for the health of their loved ones. Those reports seem to confirm the dilemma observed during the previous SARS pandemic. According to Perrin et al., medical workers save lives in their daily work, however, they are in fear of being infected or are concerned for the health of their loved ones [34].

The presented study also highlighted the fact that those health professionals who were directly seconded to fight the COVID-19 pandemic exhibited significantly greater mental strain than their colleagues. The same happened to individuals who, by the decision of national authorities or management of health facility, were seconded from their home facility to a COVID-19 unit, frequently many miles from their place of residence, resulting in separation from their families and necessary temporary change of the place of residence. Those results are consistent with the findings observed by Di Tella et al. They proved that professionals directly involved in the COVID-19 fight were significantly more likely to suffer mental health deterioration resulting from a high risk of infection, reduced personal protective equipment and, frequently, separation from loved ones, as well as the traumatic experience related to high patient mortality in the course of COVID-19 [22].

Although the presented study did not address the stigmatisation of medical personnel in relation to their work with COVID-19 patients, there was a high level of public ostracism towards medical workers among the Polish society at the time of data collection. This was reflected in, for example, physical attacks, mental harassment and general online hatred. Scientific reports clearly indicate a tremendous increase in psychotic complaints in the form of anxiety, depression, occupational burnout and mental fatigue due to stigmatisation [35]. During the pandemic period, the mass media became the main source of information and COVID-19 became one of the most searched phrases in news [36]. The analyses proved that daily tracking of both statistics and information concerning COVID-19 worsened mental health in a significant way [37]. Those results are consistent with the presented study, in which there was also a relationship between searching the web for information concerning COVID-19, as well as tracking death statistics, and mental health in the form of a comprehensive analysis of the GHQ-28 scale and its subscales of anxiety and sleep disorders, somatic symptoms and social dysfunctions.

Among health care workers, as in the general female population, people with a psychiatric history and those suffering from chronic diseases show a deterioration in their mental condition [1,38]. Their mental condition is also influenced by the daily browsing of the Internet in search of information on COVID-19 and by tracking disease and death statistics. These results are consistent with world reports, and they show that the mass media influence the creation of attitudes of both medical and non-medical workers [38,39].

As regards the potential confounding factors, it was not demonstrated that the distribution of sex, age, marital status, earning capacity during the pandemic, psychiatric history, chronic medical conditions, quarantine or COVID-19 infection in the respondent and their family members statistically significantly affected the differences between medical and non-medical professionals on the GHQ-28 scale. It does not change the fact that the aforementioned factors can significantly affect mental health, as was confirmed in previous reports [29].

The authors are aware of the limitations of this study, i.e., the method of data collection via an online survey. For this reason, it is not possible to identify the number of respondents who found out about the survey; the feedback percentage cannot be determined. Moreover, it is not possible to estimate the number of surveys that were not completed without providing any reasons at any stage. This fact may have an impact on the lowering of the final results of this study because, as earlier reports indicate, people suffering from mental disorders are less predisposed to participate in research [40,41]. It should also be noted that the surveyed group is not representative both for the Polish society and for the distribution in terms of occupation, gender, age and place of residence in the case of health care workers. Another limitation of the study is the lack of division of professions among non-medical workers. The methodological limitation of the study is the fact that only the GHQ-28 scale was used. Its analysis makes it impossible to make an unambiguous psychiatric diagnosis. For this purpose, a specialist consultation is required. Additionally, it should be noted that, due to the distribution method and complete anonymity of the questionnaire, the authors of the presented study did not have the possibility to provide psychological support to the respondents. A potential positive aspect for the participant taking part in the survey may be the fact that they may reflect more deeply on their own health and seek the services of a professional if necessary.

In conclusion, the results of this study clearly indicate the significant mental strain of medical personnel during the COVID-19 pandemic. The mentioned psychological burden was especially exacerbated in the form of anxiety, sleep disorders and somatic symptoms. This mental strain particularly concerns the health professionals who are directly involved in SARS-CoV-2-infected patient care, as well as those who were forcibly seconded to take care of such patients. The main concern for both medical and non-medical workers is the concern for the health of their loved ones. Healthcare workers show more favourable social attitudes to stop the COVID-19 pandemic in the form of reducing meetings with friends and family and keeping leaving home to a minimum. They are also more positive about the use of protective masks in both open and closed spaces, at the same time indicating a subjective deterioration of the mental condition in the time of a pandemic in nearly three quarters of medics. The above-mentioned situations frequently force them to make personal sacrifices. Since the mental health of medical personnel directly affects the results of their work, it is necessary to provide them with adequate psychological care, all the more so because the effective work of medical personnel is invaluable in the current situation [13,42,43].

## 5. Conclusions

The COVID-19 pandemic has significantly affected the deterioration of mental health in health professionals, especially those who provide direct care for SARS-CoV-2-infected patients. Health professionals show a higher level of fear for their own health and that of their loved ones. They are also much more receptive to respecting social distancing and changing daily habits to reduce the spread of SARS-CoV-2. Due to the significant mental strain, it is advisable to implement adequate psychological care for medical personnel to mitigate the effects of the pandemic. To improve the mental condition of medical workers, it is possible to use methods of mental and spiritual support, including telephone psychological support, team support sessions and art therapy, which have already been used in other countries [44]. There is a need to further assess and observe the impact of the COVID-19 pandemic on the mental health of health professionals to detect possible long-term complications. Consideration should also be given to expanding the scope of research, using tools to assess the level of occupational burnout and stress coping strategies that can enable a better understanding of the impact of the COVID-19 pandemic on the mental health of medical personnel.

## Figures and Tables

**Table 1:** Distribution of the study group according to medical/non-medical profession.

	Medical Profession (*n* = 848) [%]	Non-Medical Professions (*n* = 1302) [%]	*p*
Sex: female	736 (86.8%)	1023 (78.6%)	<0.001
Age [years]	34.14 ±8.00	32.5 ±10.09	<0.001
Place of residence: city > 250,000 inhabitants	449 (52.9%)	837 (64.3%)	<0.001
Marital status: single	160 (18.9%)	458 (35.1%)	<0.001
Level of education: higher (university degree)	782 (92.2%)	997 (76.6%)	<0.001
Limited earning capacity: no	660 (77.8%)	796 (61.1%)	<0.001
The use of psychiatric/psychological services due to the COVID-19 pandemic: yes	62 (7.3%)	95 (7.3%)	0.989
Past psychiatric treatment: yes	166 (19.6%)	208 (15.9%)	0.86
Psychiatric medication use: yes	175 (20.6%)	251 (19.3%)	0.005
Chronic conditions: yes	192 (22.6%)	294 (22.6%)	0.97
Being quarantined: yes	226 (26.7%)	196 (15.1%)	<0.001
COVID-19 diagnosis: yes	167 (19.7%)	120 (9.3%)	<0.001
COVID-19 diagnosis: yes, confirmed in a loved one	659 (77.7%)	824 (63.3%)	<0.001
COVID-19-related death of a loved one: yes	171 (20.1%)	139 (10.6%)	<0.001

**Table 2:** The comparison of mental health according to medical/non-medical profession.

Variable	Medical Professionals (*n* = 848)	Non-Medical Professionals (*n* = 1302)	*p*
GHQ-28, a positive score	545 (64.27%)	727 (55.84%)	<0.001
GHQ-28	30.35 (14.53)	28.55 (15.16)	<0.001
GHQ-28—social dysfunction	8.86 (3.40)	9.06 (3.61)	0.665
GHQ-28—severe depression	3.82 (4.36)	4.22 (4.72)	0.131
GHQ-28—somatic symptoms	7.83 (4.40)	6.70 (4.21)	<0.001
GHQ-28—anxiety and insomnia	9.85 (5.26)	8.56 (5.17)	<0.001
Fear for one’s own health	5.79 (2.30)	5.05 (5.76)	<0.001
Fear for loved ones	8.57 (1.83)	7.39 (2.85)	<0.001
Fear of being infected with COVID-19: yes	761 (89.74%)	967 (74.27%)	<0.001
Limitation of family gatherings: yes	602 (70.99%)	585 (44.93%)	<0.001
Limitation of gatherings with friends	690 (81.37%)	650 (49.92%)	<0.001
Minimised outings: yes *	709 (83.61%)	756 (58.06%)	<0.001
Staying home to reduce the spread of the pandemic: yes	289 (34.16%)	276 (21.21%)	<0.001
Wearing face masks in a confined space: yes, always/usually	847 (99.88%)	1266 (97.24%)	<0.001
Wearing face masks in an open space: yes, always/usually	823 (97.05%)	1093 (85.95%)	<0.001
Subjective deterioration of mental health: yes	613 (72.28%)	832 (63.90%)	<0.001

The results include mean values, response rate (SD; %). * I strongly agree/I agree.

**Table 3:** The assessment of the effect of work in the COVID-19 fight and secondment to fight the pandemic on the GHQ-28 scale scores, and the subjective feeling of fear.

	Work with COVID-19 Patients			Forced Secondment to Work with COVID-19 Patients		
	Yes (*n* = 561)	No (*n* = 287)	*p*	Yes * (*n* = 74)	No (*n* = 774)	*p*
GHQ-28, a positive score	69.52%	54.01%	<0.001	72.97%	63.44%	0.101
GHQ-28	32.14 (14.68)	26.85 (13.57)	<0.001	34.92 (15.91)	29.91 (14.32)	0.011
GHQ-28: social dysfunction	8.99 (3.47)	8.60 (3.27)	0.055	9.30 (3.54)	8.82 (3.39)	0.209
GHQ-28: severe depression	4.01 (4.48)	3.45 (4.12)	0.049	4.85 (5.31)	3.72 (4.25)	0.112
GHQ-28: somatic symptoms	8.52 (4.42)	6.46 (4.01)	<0.001	9.33 (4.50)	7.68 (4.36)	0.002
GHQ-28: anxiety and insomnia	10.61 (5.21)	8.34 (5.02)	<0.001	11.43 (5.22)	9.69 (5.24)	0.009
Fear for one’s own health	5.43 (2.54)	5.83 (2.28)	0.148	5.83 (2.33)	5.72 (2.24)	0.516
Fear for the health of loved ones	8.67 (1.80)	8.56 (1.83)	0.568	8.69 (1.72)	8.34 (2.01)	0.010

The results are expressed as % of responses or mean +/− SD. * Secondment by the decision of the management of health facility or national authorities.

**Table 4:** A detailed analysis of the effect of individual factors on GHQ-28 score and its subscales among medical staff.

Variable (*n* = 848)		GHQ-28		GHQ-28: Somatic Symptoms		GHQ-28: Anxiety/Sleep Disorder		GHQ-28: Social Dysfunctions		GHQ-28: Depression	
		M (SD)	*p*	M (SD)	*p*	M (SD)	*p*	M (SD)	*p*	M (SD)	*p*
Sex	Male	26.41 (12.94)	<0.001	6.45 (4.05)	<0.001	8.52 (4.94)	<0.001	8.08 (3.18)	0.001	3.33 (4.08)	0.007
	Female	30.94 (14.56)		8.03 (4.41)		10.04 (5.27)		8.97 (3.42)		3.88 (4.40)	
Place of residence	city/town > 250,000 population	30.85 (15.06)	0.756	7.83 (4.47)	0.972	9.91 (5.33)	0.893	8.99 (3.49)	0.302	4.09 (4.62)	0.483
	city/town 50,000–250,000 population	30.26 (14.29)		7.80 (4.29)		9.78 (5.19)		9.00 (3.45)		3.69 (4.24)	
	city/town of up to 50,000 population	30.10 (13.95)		8.06 (4.79)		10.01 (5.35)		8.71 (3.22)		3.30 (3.71)	
	countryside	28.71 (13.21)		7.59 (3.89)		9.51 (5.02)		8.23 (3.08)		3.39 (4.00)	
Marital status	married	29.30 (14.22)	<0.001	7.80 (4.44)	0.120	9.62 (5.20)	0.185	8.63 (3.30)	0.006	3.24 (3.90)	<0.001
	in a romantic relationship	32.55 (15.25)		7.91 (4.40)		10.81 (5.49)		9.28 (3.47)		4.55 (4.84)	
	divorced	24.25 (8.13)		7.35 (3.71)		7.80 (5.36)		6.90 (2.42)		2.15 (2.32)	
	widowed	39.00 (9.42)		9.85 (1.95)		13.00 (4.16)		11.00 (3.11)		5.14 (4.22)	
	solitude	32.32 (15.11)		7.77 (4.43)		9.74 (5.07)		9.44 (3.68)		5.38 (5.12)	
Restriction on earning opportunities	Yes, I lost my job	43.85 (15.90)	0.005	10.43 (4.36)	0.009	12.71 (5.76)	0.006	12.71 (4.07)	0.004	8.00 (5.60)	0.047
	Yes, a decrease in income ≥25%	35.80 (15.89)		9.23 (4.85)		11.74 (5.32)		10.21 (3.91)		4.60 (5.30)	
	Yes, a decrease in income ≤25%	35.85 (18.24)		9.37 (5.04)		11.61 (6.18)		9.78 (3.71)		5.09 (5.55)	
	Yes, income has remained unchanged	28.60 (12.60)		7.58 (3.98)		9.42 (5.17)		8.28 (2.89)		3.33 (3.42)	
	No	29.71 (14.21)		7.69 (4.36)		9.68 (5.17)		8.72 (3.36)		3.63 (4.22)	
	I didn’t work before or during the pandemic	28.32 (12.02)		6.11 (3.19)		8.18 (4.65)		9.00 (2.86)		5.04 (4.48)	
Psychiatrist/psychologist services during the pandemic	Yes	41.45 (13.80)	<0.001	10.85 (4.49)	<0.001	13.59 (5.22)	<0.001	10.53 (3.39)	<0.001	6.46 (5.45)	<0.001
	No	29.47 (14.25)		7.58 (4.30)		9.54 (4.10)		8.72 (3.37)		3.60 (4.18)	
The use of psychiatric medications	Yes	34.82 (15.78)	<0.001	9.20 (4.27)	<0.001	11.08 (5.24)	<0.001	9.45 (3.71)	0.022	5.08 (5.24)	<0.001
	No	29.18 (13.95)		7.46 (4.27)		9.52 (5.21)		8.70 (3.42)		3.49 (4.04)	
Past psychiatric treatment	Yes	34.78 (15.72)	<0.001	9.22 (4.67)	<0.001	10.86 (5.17)	0.006	9.52 (3.82)	0.015	5.13 (5.23)	<0.001
	No	29.26 (14.02)		7.48 (4.26)		9.59 (5.25)		8.69 (3.27)		3.49 (3.49)	
Chronic conditions, e.g., heart disease, lung disease	Yes	34.78 (15.72)	0.010	9.22 (4.67)	0.004	10.86 (5.25)	0.074	9.54 (3.82)	0.019	5.13 (5.23)	0.115
	No	29.26 (14.02)		7.48 (4.26)		9.59 (5.17)		8.69 (3.27)		3.49 (4.06)	
Being under quarantine	Yes, I am under quarantine	32.80 (15.08)	0.355	10.09 (4.93)	0.007	10.05 (5.65)	0.467	9.44 (3.03)	0.396	3.22 (4.10)	0.279
	Yes, I was under quarantine	29.39 (14.11)		7.63 (4.24)		9.40 (5.15)		8.78 (3.25)		3.58 (4.34)	
	No	30.47 (14.61)		7.73 (4.37)		9.96 (5.26)		8.84 (3.47)		3.92 (4.38)	
Recovering from COVID-19	Yes, I’m undergoing recovery from COVID-19	34.21 (14.58)	0.129	11.39 (4.90)	<0.001	10.26 (5.98)	0.749	9.65 (3.17)	0.075	2.91 (3.44)	0.180
	Yes, I recovered from COVID-19	29.64 (15.17)		7.95 (4.60)		9.64 (5.53)		8.52 (3.51)		3.52 (4.27)	
	No	30.21 (14.39)		7.56 (4.22)		9.85 (5.16)		8.86 (3.39)		3.92 (4.42)	
COVID-19 confirmed in a family member/close friend	Yes	30.79 (14.83)	0.076	8.05 (4.37)	0.002	9.97 (5.21)	0.161	8.92 (3.46)	0.301	3.83 (4.35)	0.363
	No	28.79 (14.60)		7.03 (4.38)		9.39 (5.39)		8.64 (3.17)		3.74 (4.44)	
COVID-19-related death	Yes, a family member	34.93 (15.45)	0.101	9.14 (4.56)	0.063	10.53 (5.41)	0.105	9.70 (3.01)	0.021	5.53 (5.38)	0.051
	Yes, in a close friend	30.75 (14.81)		8.21 (4.49)		10.62 (5.12)		8.58 (3.69)		3.32 (3.93)	
	No	29.99 (14.38)		7.67 (4.35)		9.65 (5.26)		8.86 (3.36)		3.80 (4.35)	
Information retrieval	Yes	31.10 (14.24)	<0.001	8.12 (4.39)	<0.001	10.23 (5.18)	<0.001	8.98 (3.41)	0.020	3.75 (4.20)	0.684
	No	27.90 (15.14)		6.84 (4.28)		8.59 (5.31)		8.46 (3.53)		4.00 (4.82)	
Statistics tracking	Yes	31.16 (14.38)	0.009	8.10 (4.45)	0.010	10.25 (5.17)	<0.001	8.94 (3.43)	0.191	3.85 (4.26)	0.180
	No	28.72 (14.71)		7.27 (4.24)		9.01 (5.33)		8.69 (3.34)		3.72 (4.55)	

## Data Availability

The data presented in this study are available on request from the corresponding author.

## Supplementary Materials

The following are available online at https://www.mdpi.com/article/10.3390/ijerph18116101/s1.
